# USABILITY OF AN ONLINE DISCUSSION PLATFORM: DEVELOPMENT OF A CODING SCHEME

**DOI:** 10.1093/geroni/igz038.3203

**Published:** 2019-11-08

**Authors:** Soojeong Han, Andrew Teng, Shih-Yin Lin, Frances Chu, George Demiris, Oleg Zaslavsky, Annie T Chen

**Affiliations:** 1 University of Washington, Seattle, Washington, United States; 2 NYU Rory Meyers College of Nursing, New York, New York, United States; 3 University of Pennsylvania School of Nursing, Philadelphia, Pennsylvania, United States

## Abstract

Online discussion platforms have the potential to support or even improve older adults’ well-being. Nonetheless, potential health benefits are often shaped by the usability of such platforms. Improving usability is imperative to maximize these potential benefits. As a part of a qualitative analysis of a study of an online community for older adults with frailty symptoms, we developed and refined a coding scheme targeting usability. The scheme was derived by reviewing contemporary literature on user experience, usability, and health information technology in older adults. Our review revealed challenges to apply commonly used terms to summarize our qualitative data. For example, the concept of perceived usability has different meanings and definitions in existing frameworks as they pertain to user engagement and technology adoption (specifically, the Technology Acceptance Model) than usability in an online discussion context for older adults. Because none of the meanings of usability fully encompassed the breadth of this concept for older users, we developed a coding scheme that is practical and captures a broad range of older adults’ perceptions of usability. Through qualitative analysis of the online discussion content using the newly developed coding scheme, new themes emerged such as confusing layout (e.g., difficulty in locating discussion boxes), insufficient instruction or training (trouble posting discussions), unwanted results (e.g., pressed a wrong key), and memory issues and cognitive burden. This presentation describes the process of developing a coding scheme, illustrates nuances of meanings in concepts related to usability, and presents preliminary results of our qualitative analysis.

